# Menstrual Health Experiences of People with Intellectual Disabilities and Their Caregivers during Vanuatu’s Humanitarian Responses: A Qualitative Study

**DOI:** 10.3390/ijerph192114540

**Published:** 2022-11-05

**Authors:** Jane Wilbur, Relvie Poilapa, Chloe Morrison

**Affiliations:** 1 London School of Hygiene & Tropical Medicine, International Centre for Evidence in Disability, London WC1E 7HT, UK; 2 World Vision Vanuatu, Port Vila P.O. Box 247, Vanuatu

**Keywords:** humanitarian responses, emergencies, menstrual health, intellectual disability, caregivers, Vanuatu, water, sanitation and hygiene

## Abstract

Attention to menstrual health in humanitarian responses is increasing, but evidence related to people with intellectual disabilities and their caregivers is absent. This study begins to address that. We applied purposive sampling to select 17 women and girls (aged 15–31) with intellectual disabilities, their 17 caregivers in SANMA province, Vanuatu, and seven key informants. We used in-depth interviews, PhotoVoice and ranking, and observation and analysed data thematically using Nvivo 12. We found that caregivers wished to maintain the person’s safety and privacy, especially when menstruating, which reduced evacuation options. People with intellectual disabilities support requirements sometimes increased after emergencies. This meant caregivers were less able to work and recover from disasters. Caregivers requested the distribution of more reusable menstrual materials and a greater choice, including adult-sized diapers for menstruation and incontinence. Key informants noted that menstrual health interventions must always be delivered to people with intellectual disabilities and their caregivers so that menstrual health knowledge and practices exist before emergencies. We found that men and women supported people with intellectual disabilities’ menstrual health, thus challenging gendered assumptions about caregiving. Efforts to achieve menstrual health for this population within disaster preparedness plans must be included. If not, families will fall further into poverty every time a disaster hits Vanuatu.

## 1. Introduction

### 1.1. Menstrual Health for People with Disabilities

Achieving menstrual health means that all women and girls who menstruate have accurate information about the menstrual cycle and how to manage it hygienically. It means everyone can access effective and affordable menstrual materials, water, sanitation, and hygiene (WASH) services. These enable people to change their material safely and privately, wash their bodies and material used, and dispose of used menstrual materials [1]. Menstrual health also involves access to medical support for menstrual-related disorders and discomfort, including pain relief, an environment free from menstrual-related stigma, and the ability to participate fully in daily life [1]. 

A range of evidence shows that many women and girls living in low-and middle-income countries (LMICs) do not have menstrual health, which negatively impacts their psychological and physical health, education, social participation, and employment [2]. A growing body of data reveals that people with disabilities face additional challenges in realising their menstrual health in LMICs. This issue is particularly evident for people with intellectual disabilities who often rely on caregivers [3,4,5,6,7,8,9,10]. For instance, a recent study in Vanuatu highlighted that women and girls must independently collect their own water, bathe, and wash their reusable menstrual materials when menstruating. Everyone found this challenging, particularly women and girls with disabilities who require support to complete these tasks but must manage alone [7]. Furthermore, women and girls with intellectual disabilities are particularly disadvantaged when menstruating [6]. Evidence from different settings demonstrates that management strategies applied by caregivers include isolating and physically restraining people with intellectual disabilities during menstruation and, in some cases, sterilisation to cease menstruation [5,6,8,9,10].

### 1.2. Menstrual Health for Women and Girls with Disabilities during Emergencies 

The recognition that menstrual health is vital to humanitarian responses is growing, with guidelines, such as the Sphere Handbook and a Toolkit that support the implementation of menstrual health in emergencies [11,12,13,14]. Women and girls with disabilities must benefit from these interventions. Yet, a recent systematised review exploring the inclusion of disability within menstrual health humanitarian responses highlighted minimal data on this; that women and girls with disabilities seldom participated in interventions, and efforts made invariably focused on the provision of accessible WASH infrastructure. The latter is only part of menstrual health [15]. These limited considerations have not yet extended to women and girls with intellectual disabilities and their caregivers, who require more holistic, customised support [6,7,8,16,17,18,19]. 

### 1.3. Inclusion of People with Disabilities in Humanitarian Responses Delivered in Vanuatu 

Vanuatu, located in the Pacific Region, is a collection of approximately 83 islands ranked 140 out of 189 countries and territories on the Human Development Index [20]. Vanuatu is ranked globally as the most at-risk country for natural hazards on the World Risk Index [21]. It is highly vulnerable to flooding, cyclones, earthquakes and volcanic eruptions [22]. For instance, in April 2020, category five Tropical Cyclone Harold made landfall on Espiritu Santo Island whilst the country was in national lockdown during the COVID-19 pandemic [22]. More than 159,000 people were affected, and many schools, medical facilities, food crops, and water supplies were severely damaged [23]. Only two years earlier, the Ambae volcano erupted for a second time (previously in 2017), evacuating the entire Ambae Island population (11,700 people) to neighbouring islands [24]. 

The National Disaster Management Office (NDMO) within the government coordinates emergency preparedness and responses in Vanuatu [25]. Within this, the Vanuatu Humanitarian Team is a network of humanitarian agencies, including non-government organisations (e.g., World Vision, Save the Children, Care, ADRA, Oxfam), United Nations Agencies (e.g., UNDP, UN Women, UNICEF), and the Secretariat of the Pacific Community. The latter supports 27 countries and territories with research and technical solutions for sustainable development. The Gender & Protection Cluster, led by the Department of Women’s Affairs in collaboration with CARE and Save the Children, was formed under the NDMO in 2014 when Tropical Cyclone Lusi hit Vanuatu. This Cluster aims to mainstream gender and protection into all humanitarian activities, identify gaps, coordinate agencies to share information and learning, and respond to those gaps.

In this vein, a study was conducted after Tropical Cyclone Pam (which hit Vanuatu in March 2015) to support agencies and communities in Vanuatu to learn lessons from Cyclone Pam and improve disability inclusion in future disaster preparedness plans and responses [26]. The study found that people with disabilities were nearly three times more likely to be injured during the cyclone than people without disabilities; men with disabilities were twice as likely to be injured than women with disabilities. Adults with disabilities had less access to humanitarian response activities than adults without disabilities, which was worse for women than men with disabilities. Women with disabilities reported less access to toilets than men with disabilities; few people had assistive devices (e.g., wheelchairs, glasses, walking canes) they needed, and some devices were lost during the cyclone. Finally, people with disabilities reported worse wellbeing than people without disabilities. Though the study did not explore gender-based violence, the authors concluded that ‘women with disabilities probably experience[d] heightened levels of violence following the disaster’ and that this could have impacted their wellbeing. 

Progress has been made regarding incorporating menstrual health in Vanuatu’s humanitarian responses. For instance, a study of menstrual health in the Pacific region reported that the humanitarian response to Tropical Cyclone Harold was a ‘turning point’ for supporting menstrual health during emergencies [27]. This was because humanitarian agencies and Government Ministries and Departments worked to strengthen knowledge about menstrual health and distribute hygiene kits that contained reusable menstrual products. Encouragingly, a growing number of studies which aim to assess menstrual health within Vanuatu’s humanitarian responses or improve them include the perspectives of women and girls with disabilities [27,28]. Both studies note that women and girls with disabilities face broader challenges managing menstruation than those without disabilities. Downing et al. [28] also highlight that some women and girls with disabilities may be unable to reach distribution sites, so they require home visits; latrines must be made accessible, and this group may have specific requirements for menstrual materials, which must be addressed in humanitarian responses. However, these studies did not explore the different challenges women and girls with intellectual disabilities and their caregivers faced. 

### 1.4. Study Aim and Research Questions

This study aimed to explore the menstrual health experiences of women and girls with intellectual disabilities (hereafter referred to as ‘young people’ or ‘young person’) and their caregivers during humanitarian crises in Vanuatu. It met this aim by asking the following research questions: how do emergencies (1) impact young people’s ability to achieve menstrual health, and (2) the ability of caregivers to support this? 

To the best of the author’s knowledge, this is the first study which explores these issues.

## 2. Materials and Methods

### 2.1. Study Site

The study was conducted in the SANMA province, in the northern part of Vanuatu. Most, if not all, people living in SANMA province have been affected by emergencies. For instance, 8385 people were evacuated to the province during the Ambae volcano eruption in 2017, and in 2020 Tropical Cyclone Harold made landfall on Espiritu Santo, affecting the SANMA province population and infrastructure [29]. 

### 2.2. Study Population and Sampling

We selected 17 young people aged 15–31 who menstruate; experience ‘a lot of difficulty or more’ across one or more of the functional domains in the Washington Group Short Set questions (visual, hearing, mobility, cognition, self-care, and communication) [30]. We also selected 17 caregivers who support the young person’s menstrual health. These caregivers were interviewed about how they support the young person’s menstrual health and their interpretation of the young person’s menstrual-related experiences and feelings. We also selected seven humanitarian actors working in Vanuatu. Table 1 presents details of the study population characteristics. 

We identified young people from lists provided by World Vision and the Vanuatu Society for People with Disability (VSPD), a national disability service provider. These lists captured the young people’s names, self-reported impairment, age, geographical location, and their caregiver’s names, geographic location, and telephone numbers. Snowball sampling (in which the research team and participants identify other potential participants) was also applied [31]. 

We applied a stratified purposive sampling [31] to select key informants to represent variation in organisations (international government organisations, non-government organisations, private sector), sectors (disability, WASH, and health), and humanitarian response (e.g., preparedness, emergency relief, early recovery, medium to long-term recovery, and community development). 

### 2.3. Data Collection Methods 

We applied three qualitative methods to enable methods triangulation: in-depth interviews, PhotoVoice and ranking, and observation. Supplementary Materials 1–3 contain the in-depth interview topic guide for caregivers, PhotoVoice and ranking guidance, and observation instructions for young people. 

We explored the following topics with key informants during in-depth interviews: the inclusion of menstrual health for women and girls with disabilities in the organisation’s humanitarian responses, available guidance and support for this, levels of awareness and understanding of disability and menstrual health, and the operational context. 

During in-depth interviews with caregivers, we explored their ability to support the young person’s menstrual health during humanitarian emergencies, the caregiver’s knowledge and understanding of menstruation, menstrual materials used by the young person, where these are disposed of or reuse practices; where the young person bathes during menstruation, how the caregiver prepares for the young person’s menstruation, any support or guidance they have received to carry out this role, and any socio-cultural beliefs related to menstruation followed. 

PhotoVoice is a visual research methodology applied in many settings to explore sensitive issues related to WASH, including menstrual health, perimenopause, and incontinence [7,32,33,34,35,36]. Participants own the photos and identify how and where to share their pictures. In our study, we conducted PhotoVoice with three caregivers; each was loaned a smartphone (with a camera) and asked to take a series of photos representing their experiences supporting the young person’s menstrual health during emergencies. We then interviewed participants to understand why they took the photos before asking the caregiver to rank them according to which images represent the most critical issue to the least. PhotoVoice was an appropriate method for this study and sample population, especially because two participants were men. This method may have allowed men to convey their thoughts and insights about this sensitive and often taboo topic more candidly than verbally in an interview with a female researcher. 

We used a doll to explore how young people understand menstrual health and their feelings. The dolls drew on the Bishesta Campaign, a menstrual health intervention for people with intellectual disabilities and their caregivers in Nepal [16]. Within the campaign, a large doll with removable clothes, underwear and menstrual materials is used to teach young people about menstrual health and increase communication about menstruation between the young person and their caregivers. To explore the menstrual health practices followed by the young person, we introduced the doll to them. Six young people engaged with the doll, so we completed the following steps during their interview:

- Showed the young person that the doll was wearing a used menstrual material by taking it out of the doll’s underwear. Passed it to the young person and observed her reaction to it (e.g., awareness, unperturbed, disgust)
- Asked the young person where the doll should put the used menstrual material and observe her response (e.g., put it in the bin, in the laundry, on the floor, throw it in the bushes)
- Asked the young person what the doll should do next and then showed her a clean menstrual material. We observed her reactions throughout, including if she indicated that the doll should put the clean menstrual material in her underwear, get dressed, and wash her hands

After the interview, the researchers discussed their observations and made field notes. All interviews were conducted in Bislama, recorded on a voice recorder, and completed in one hour. As interviews were conducted during COVID-19, the government recommended that preventative measures be adhered to. 

### 2.4. Data Analyses

Data were analysed iteratively. The research team met twice a week during data collection to discuss emerging findings and themes (e.g., the need to distribute more menstrual materials in hygiene kits during humanitarian responses) to ensure these were explored in future interviews. Recordings of interviews were translated into English and transcribed during data collection so data could be analysed concurrently. All identifiers were removed from transcriptions and observation field notes to ensure participant anonymity. Bislama-speaking team members checked the accuracy of transcriptions and made revisions if required before finalisation. 

Two authors thematically analysed transcriptions by following this process: (1) familiarisation with data, (2) first-level coding using pre-determined codes that reflected issues investigated in the topic guides (e.g., humanitarian assistance, water, sanitation, and hygiene services used for menstrual health), (3) identifying additional codes, (4) developing an analytical codebook to organise data, (5) double-coding a small proportion of data to improve codebook content [37], (6) coding all transcripts (7) reviewing relationships between codes and analyses with the broader research team. We organised data and captured analyses using Nvivo 12.

### 2.5. Informed Consent Process

Informed consent was sought from every participant before enrolment. Information and consent sheets (in Bislama) were read to the participants by the researchers. Written (or thumbprint if illiterate) informed consent was obtained from all caregivers and key informants. Assent was sought from all young people, and consent was sought from their caregivers.

The informed consent process for PhotoVoice was twofold. Firstly, written consent was sought at the initial meeting when the task was explained, including how to take photos without showing faces. Secondly, written informed consent was sought when the participant had taken the photos, had been interviewed, and ranked the images. At this point, they could better state how to use the photos. All participants were asked if they wanted to be credited by their real name or a pseudonym, which they picked. 

Researchers made it clear that people’s participation was voluntary, and they could withdraw at any time without stating a reason. Researchers explained that this would not harm future relationships between the participants or the organisations they represent. 

### 2.6. Research Team 

The research team consisted of an academic from the London School of Hygiene & Tropical Medicine (who remained in the UK for the study), Ni-Vanuatu women with and without disabilities working for World Vision and VSPD and as an independent consultant. 

## 3. Results

In summary, the results are presented across the following themes: ‘Participation and safeguarding’ during menstruation in emergencies and non-humanitarian contexts, ‘Decisions during emergencies’, which are partly driven by safeguarding concerns; families ‘Resilience to disasters’, caregivers and key informants’ feedback on ‘Hygiene kit content’; ‘Menstrual health interventions in non-humanitarian settings’ and their importance in successfully promoting menstrual health interventions during emergencies, ‘The importance of WASH service for menstrual health’, ‘Menstrual health caregiving roles and impacts’, and finally, ‘Observation of young people’.

### 3.1. Participation and Safeguarding

Fear and experience of gender-based violence mean parents either keep their daughters with intellectual disabilities at home or always stay with them when they go outside. Therefore, few reported changes in participation when menstruating because this was generally curtailed. However, almost half of the caregivers reported keeping the young person at home when menstruating because she may leak menstrual blood on her clothes or take her used menstrual material out in front of others.

*“Sometimes, she messes on her clothing; that’s why I don’t want her to go out of the house when she menstruates. She can go out when the menstruation is over. If she happens to go out when she menstruates and messes on her clothes, I am embarrassed when people come to tell me.”*

(Female caregiver)

*“She removed [her menstrual material] and just threw it down. When her brothers saw it, they called out to the woman and said, “Come and pick up this little girl’s dirty [menstrual] cloths.” Because she didn’t know what to do because I wasn’t there. She pulled it out then threw it down and walked around.”*

(Female caregiver)

The need to stay with the young person, especially when she is menstruating, impacts the caregiver’s ability to participate fully in daily life.

*“When she menstruates, I stay with her at home. I cannot leave her alone at home. I have to be with her all the time until her menstruation is over.”*

(Female caregiver)

Many caregivers could not earn an income when supporting the young person during menstruation.

*“Since I have a daughter whose situation is like this, it makes life difficult for me. I don’t sell every day because I need to be with her if she has a problem or if she is sick [menstruating].”*

(Female caregiver)

*“I call my boss and ask to take leave for three days—I explain to him that my daughter is like this, and then he agrees. I stay home until her period is finished, and then I go back to work.”*

(Female caregiver)

**Figure 1:**
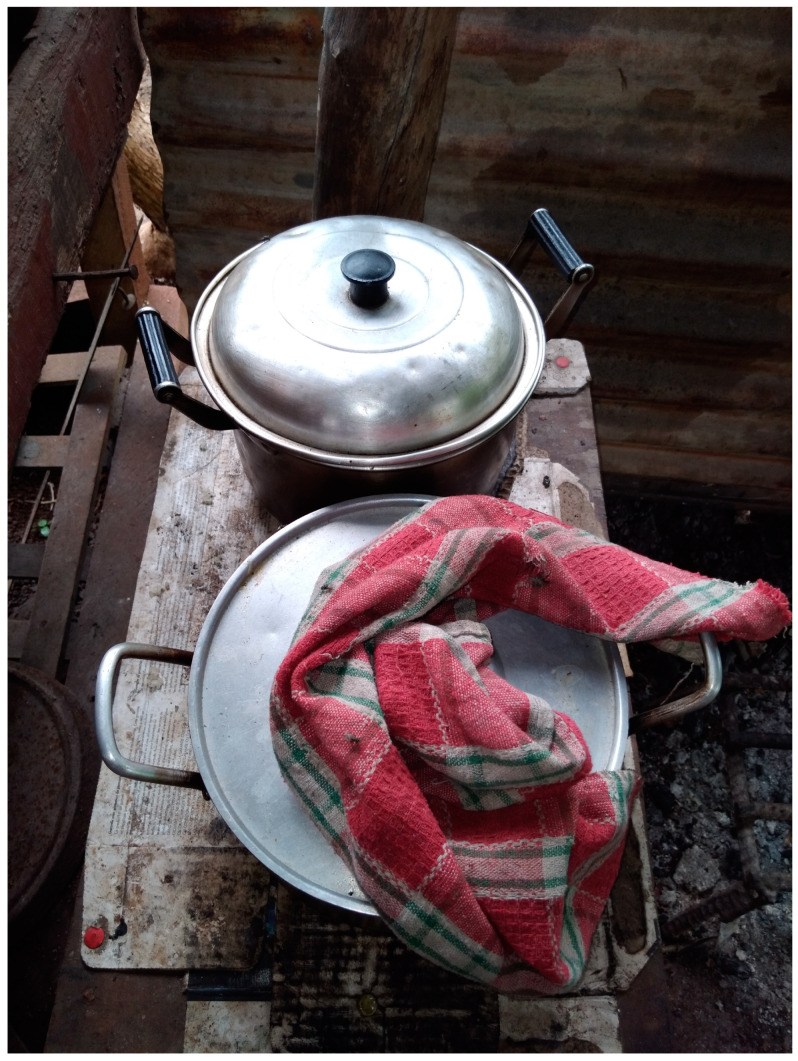
Unable to generate an income. **Photo caption:** She prefers to wear disposable pads now but needs money for that. She wants to be able to earn money from selling food, but she struggles to cook food in order to earn money. **Photo credit:** Tony.

The impact of disability on young people’s potential to earn an income was highlighted by a caregiver who took part in PhotoVoice (Figure 1). Tony’s photo and caption (Figure 1) indicate how few income-generation activities are possible for young people and speak to disability discrimination in employment.

### 3.2. Decisions during Emergencies

Many caregivers worried about the safety of their young person if they left home or were at home alone; in some cases, this was based on experience. The following quote is given by a mother of a young person who was sexually abused. The mother explained that her daughter does not allow anyone to massage her stomach to relieve menstrual discomfort because she fears being touched.

*“Sometimes we would hear that when she’s here [alone], men would come and play with her.”*

(Female caregiver)

Caregivers spoke about how people stay in caves, with neighbours and family members, or at evacuation centres after an emergency. They noted that these places are often crowded, so safeguarding concerns, a desire for privacy, and disability discrimination significantly influenced caregivers’ choices about where they evacuated.

*“We couldn’t go elsewhere because nobody likes her.....We do not go to other people’s houses. […] The three of us didn’t go anywhere. We took shelter inside our kitchen. The wind blew out part of the kitchen.”*

(Female caregiver)

**Figure 2:**
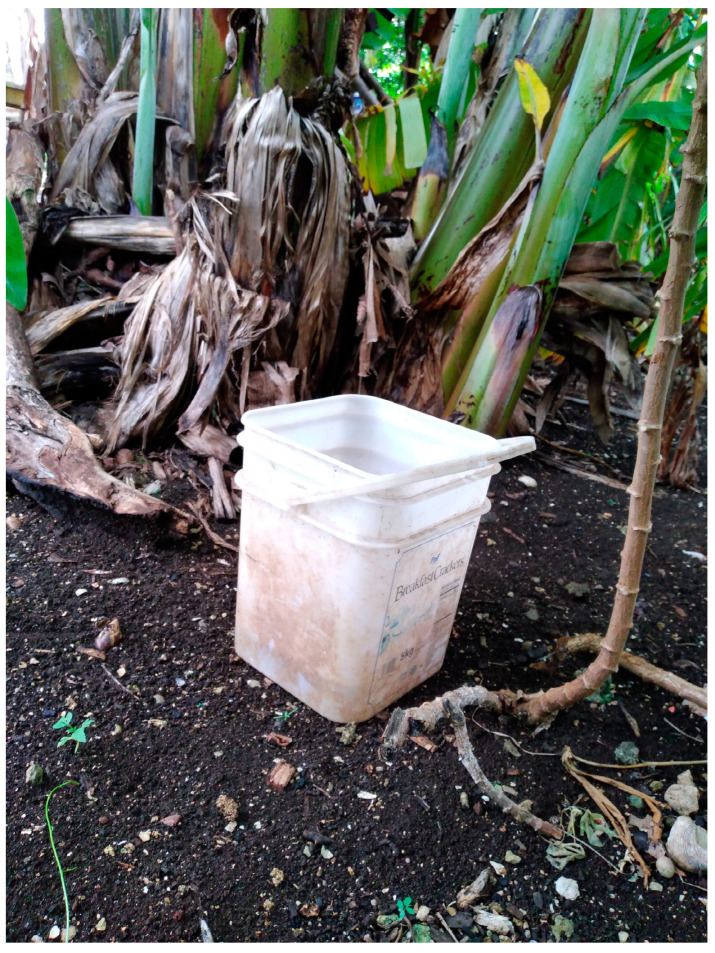
No privacy. **Photo caption:** In [our uncle’s] house, there is no privacy. Therefore, when she is dirty [menstruates], I had to take her out of that place, away from other people and come here where we feel that there is privacy. I would fetch a bucket of water and put it behind a tree where no one could see her when she was having her shower. There was hardly any tree standing, but I had to find somewhere she could shower so no one could see her. **Photo credit:** Josephine.

One caregiver who took part in PhotoVoice took an image (Figure 2) which shows where Josephine took her daughter to bathe in the aftermath of Tropical Cyclone Harold. Josephine explained that she thought the banana tree gave her daughter more privacy than the house they evacuated to. 

Several key informants also noted how a lack of privacy and safety at evacuation centres influences the choices of families with women with intellectual disabilities.

*“Even though they don’t have a house anymore or it is damaged, these victims will not want to move into the evacuation centres. […] I have seen at the evacuation centre who needed privacy […]. Carers are also ashamed because they are with these young girls who need to use the toilets and are menstruating.”*

(Key informant)

When asked what the priority should be for this population during emergencies, another key informant drew on their experiences of the Ambae evacuation and answered that evacuation centres should have private spaces for these families. However, the participant also noted that the situation had not improved during Tropical Cyclone Harold.

*“They had houses which were totally damaged, they had no food, and their places were muddy. They could not go anywhere, so they had to evacuate to some churches and classrooms, but then there was no privacy.”*

(Key informant)

Other key informants noted that privacy is an important issue, but they could not identify how to improve this.

*“I don’t know how because these are church buildings and church compounds, but to find a way to be able to accommodate people with intellectual issues and have privacy for themselves and their carer. [….] Because when this is not made available […] even though they don’t have a house anymore or it is damaged, these victims will not want to move into the evacuation centres.”*

(Key informant)

### 3.3. Resilience to Disasters

**Figure 3:**
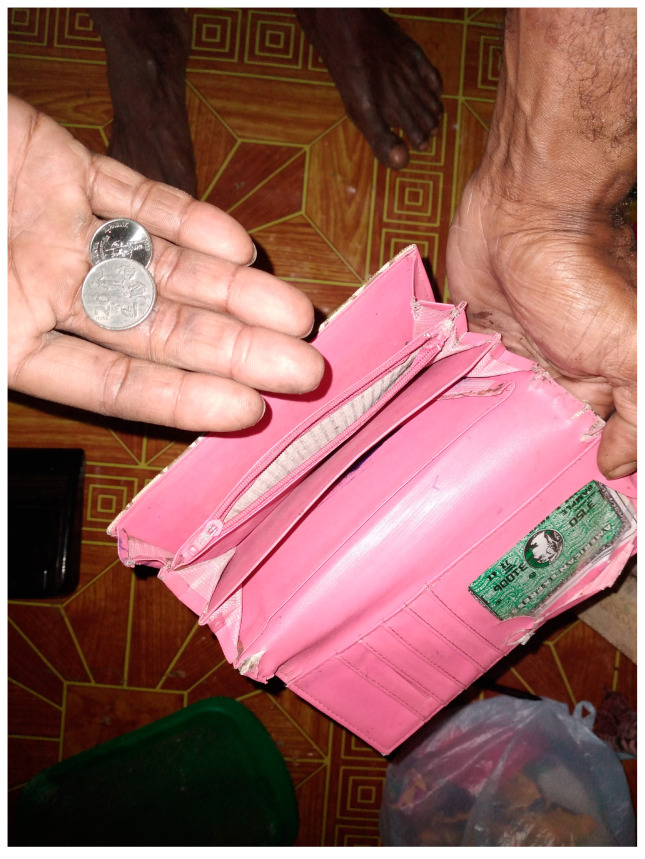
Ceasing employment to provide care. **Photo caption:** “My wife and I decided that I would no longer work and that I would prioritise looking after the children and my sister. However, this has not only affected my nuclear family but also my mum and dad because they have the same needs as [my sister]—diapers and pads”. **Photo credit:** Nick.

Many caregivers spoke about their precarious financial position before emergencies. This often related to the need for caregivers to provide full-time support to the young person, meaning their earning potential is reduced. For instance, Nick, who took part in PhotoVoice, explained that he gave up work to look after his sister, who has intellectual disabilities (Figure 3). He took a photo of the purse with a few coins to demonstrate the family’s income poverty.

Another caregiver explained that they have less time to work because the young person’s health is declining. They also said how an inability to earn money made it harder to rebuild their homes post-cyclone.

*“You had asked a question of how we are earning an income, and we said it was through kava, but in reality, we hardly have time to plant kava now that her situation is deteriorating. […..] We need to rebuild, but we cannot do that because everything is money.”*

(Female caregiver)

### 3.4. Hygiene Kit Content

Caregivers reflected on the content of hygiene kits they received in Tropical Cyclone Harold and for any recommendations, they had for kit content and delivery in future emergencies. Contents distributed in hygiene kits were menstrual materials (commercial and reusables), underwear, child diapers, soap and laundry detergent, toothpaste and toothbrushes, small and large towels, and a bucket. Many caregivers expressed appreciation for these.

*“They have included everything we needed. We were so happy with the things we received. […] I feel so fortunate because I don’t have to buy pads anymore. We have received free pads.”*

(Female caregiver)

When asked how kits could be improved, most caregivers said they would like a greater choice of menstrual products to meet specific requirements. For instance, several young people who menstruate also experience incontinence, and many wanted diapers as these could be used for incontinence and menstruation. They felt this is particularly important post-emergency because stress increased the regularity of incontinence.

P: Before the cyclone, [incontinence] wasn’t too much but after the cyclone…

I: She misses going to the toilet more than before the cyclone came?

P: Yes

One caregiver explained that she prefers her daughter to use diapers when menstruating, though these are unaffordable. She was pleased to receive these in the hygiene kits and washed, dried, and reused them as they were valuable. However, she could not do this quickly enough because it was raining heavily after the cyclone. Hence, the caregiver recommended distributing more menstrual health materials in the kits.

*“…with the rain, it is not enough. When it rains, six is not enough. I’d wash these ones, but the others would not be dry yet.”*

(Female caregiver)

Key informants also noted that more menstrual materials need to be distributed to families that include people with disabilities.

*“I think that where there is a hygiene kit, you know, one pack of menstrual pads to a family. That is not enough.”*

(Key informant)

Key informants highlighted the need to be better prepared for the next emergency and recommended storing more hygiene kits for distribution during the next crisis.

*“Wouldn’t it be great if we could have a team doing pre-positioning […] and having the products on-site. Because we know there’s gonna be some sort of natural disaster, whether it be a tsunami, earthquake or cyclone. It’s going to happen in Vanuatu.”*

(Key informant)

### 3.5. Menstrual Health Interventions in Non-Humanitarian Settings

Most key informants highlighted the need to deliver menstrual health interventions in non-emergency settings and during rapid humanitarian responses. For instance, key informants explained that the drive to distribute hygiene kits quickly during emergencies means little or no time to combat harmful menstrual taboos, which is an essential component of menstrual health.

*“What I see is happening is […]: “here’s your hygiene kit; we move on to the next village now”. So, and you know it’s a really sensitive topic, so clearly someone needs to take charge in that village to be responsible for education and the dispersal of the education that goes alongside the kit. And so often a reusable product is possibly a new product, and there is no knowledge about how do you use it.”*

(Key informant)

Many key informants noted the importance of delivering menstrual health campaigns before emergencies so that awareness about menstrual health is already present. Therefore, people may be more likely to use menstrual materials distributed.

*“The importance of when a new product is issued in a hygiene kit, that it is vital that the people distributing have got the knowledge [about] how to use it. The anecdotal information I had is that there were children running around with reusable menstrual pads on their arms as wings, playing.”*

(Key informant)

Furthermore, key informants noted that the information provided about using menstrual materials in the hygiene kits was too complex and detailed for everyone, including those with limited literacy and education, to understand.

*“I think the briefing card was too detailed. It was too wordy, and when I read it and tried to transfer it down to a level where they could understand, it was too much in a short time.”*

(Key informant)

Many key informants noted the importance of delivering menstrual health interventions for women and girls with disabilities before emergencies because of the additional challenges this population faces when menstruating.

*“Women and girls that have a disability in peacetime still face many challenges in managing menstrual health. They do not feel comfortable speaking about it. […] They will feel shy, or they will fear talking about their needs, so they really isolate themselves.”*

(Key informant)

Key informants also highlighted the need to develop networks with disability service providers and Organisations of Persons with Disabilities and relationships with people with disabilities and their families before emergencies, as this would enhance the responder’s ability to deliver what is required.

*“If we have that specific initiative available [for people with disabilities] during peacetime, we are working with them, we understand them, we are creating a relationship. […] Because when it comes to disaster, and we are making quick decisions, it’s very easy to overlook the need of persons with disabilities.”*

(Key informant)

### 3.6. The Importance of WASH Services for Menstrual Health

Key informants noted the importance of providing water services within menstrual health efforts.

*“It is all right to give them calico, but we need to think of water before we give because if we give and there is no water, that is a big problem.”*

(Key informant)

The impact of having inadequate sustainable WASH services was keenly felt by participants with disabilities and their caregivers during emergencies, especially during menstruation when people want to bathe more often.

*“It was really hard, we tried really hard. Sometimes we take a truck to the sea [to bathe]. […] So if you have money, you must have money because it costs to take the truck down to the sea.”*

(Female caregiver)

### 3.7. Menstrual Health Caregiving Roles and Impacts

In several households, menstrual care support was provided by women, but not in all. Sometimes men share the role with women; in one case, a man offered sole support for his female family member and explained why.

*“Since they cut mummy’s leg and daddy is now sick and down in bed, I have no choice but to help her because I love her so much. That’s why I have to take care of her because if my wife did it, she would complain.”*

(Male caregiver)

However, societal assumptions that women provide care, especially during menstruation, are prevalent. These viewpoints were revealed through interactions between researchers and participants during interviews. For instance, a key informant said that “men do not think about these things” (menstrual care). The following excerpt from an in-depth interview shows how surprised the researcher was when she was told that the young person’s father supports her in managing menstruation:

I: Excuse me, I just want to ask a question: Does her father help to take care of her?

P: Yes, sometimes, if I’m out, her father will help her change [her menstrual material] when she has her period.

I: Her father? He helps?

P: Yes

The wife of one man who provided menstrual care for their daughter explained how some people ostracise her husband because of this role. This reveals the impacts of menstrual socio-cultural beliefs held by many.

*“Whenever there is community work, my husband would bring cooked food to share with the others during mealtimes. However, none would want to eat the food he brings with him [because he supports her during menstruation].”*

(Female caregiver)

Many caregivers discussed the challenges of supporting people with intellectual disabilities.

*“I tell him that he should make it his duty to take care of her since he was the one who assisted in making sure that she was alive. If he hadn’t done that, we would not be burdened in taking care of her now.”*

(Female caregiver)

Yet, one caregiver, an educated professional, spoke about the joy this role can bring.

*“I’m doing this because children such as these bring blessings, lots of blessings that support those of us who are able-bodied.”*

(Female caregiver)

### 3.8. Observation of Young People

Many young people who were initially shy or reserved with the researchers became more engaged when they were shown the doll. Therefore, the researchers could observe their behaviour and write notes on these exchanges. The following field notes capture one young person’s reaction to the doll.

*“When I brought out the doll, she smiled widely, and her eyes brightened. The doll really got her attention.”*

(Field notes)

Many young people did not want to touch the doll’s used menstrual material, but some indicated an awareness about how to dispose of them, as recorded in these field notes:

*“The doll was the one that made her sit up straight and be more focused during the rest of the interview. She [young person] didn’t want to touch the used pad but pointed to the bucket to indicate where the used pad should go.”*

(Field notes)

One young person said that the doll must bathe when menstruating. Another participant needed a lot of encouragement from her caregiver to change and dispose of the doll’s menstrual material, thus indicating limited confidence in carrying out the tasks. The researcher discussed this assumption with the young person’s caregiver, who confirmed it. 

## 4. Discussion

This study explores the menstrual health experiences of young people with intellectual disabilities and their caregivers during humanitarian emergencies in Vanuatu. We noted several key findings that will be discussed in relation to existing evidence to demonstrate how our study contributes to the discourse topic.

Evidence about how menstruation curtails the participation of women and girls is far-reaching. Equivalent data for women and girls with disabilities are also growing. For instance, the Joint Monitoring Programme reports that this population is 50% less likely to participate in daily life than women and girls without disabilities [38]. A previous study we conducted in Vanuatu to explore and compare the menstrual health experiences of people with and without disabilities noted that those with disabilities were more likely to eat alone and almost two times more likely to miss out on social activities [7]. We also noted that caregivers of people with intellectual disabilities tended to keep the person at home when menstruating because of the risk that menstrual blood would soak onto their clothes or that they would take their menstrual material off in front of others [7]. These findings are also reflected in studies from Nepal, India, Australia, and New Zealand [8,18,39]. 

We noted similar concerns in this study, but we also found that previous experience of, or fear of, the risk of gender-based violence, including sexual violence, drove caregivers to keep young people inside the home or accompanied most of the time, particularly during menstruation. During and after emergencies, the desire to protect the young person and maintain privacy when she is menstruating influenced the caregiver’s decisions about where to evacuate. Consequently, support must be provided to young people and their families to evacuate to safe and private rooms or buildings. As poor access to WASH impacts people with disabilities more acutely than people without disabilities [7,34,40], the provision of sustainable WASH services before, during, and after emergencies are equally crucial for menstrual health, privacy, and safety. 

These findings reveal how discrimination intersects gender, disability, and menstruation and negatively impacts the ability of women and girls with intellectual disabilities to participate in daily life fully. The impacts are also felt by caregivers, who stay at home with the young people, thus reducing their potential to earn an income or risk losing gainful employment, which is worrying and, therefore, can negatively affect mental health. Furthermore, young people’s support needs may increase post-emergencies, meaning that caregivers are less able to work when income generation is significant for recovery. During humanitarian responses, people with disabilities and their families must be targeted with humanitarian assistance and hygiene kits, which include menstrual materials, so they do not have to pay for them. This cycle of income poverty, declining mental health, and reduced ability to work push families further into poverty every time another natural disaster occurs. Globally, evidence emphasises that people with disabilities are more likely to live in disaster-prone locations and are repeatedly excluded from disaster preparedness plans [41,42,43]. Our study provides more granular evidence, but the impact of climate risks and hazards on poverty, health, and wellbeing for people with and without disabilities is required. 

Our findings report that the incontinence some young people experienced worsened due to trauma experienced during and after the cyclone. As people with disabilities are three times more likely to experience incontinence than people without disabilities in Vanuatu [34], a variety of menstrual materials, including adult-sized diapers that can be used for incontinence and are usually unaffordable, must be included in hygiene kits. More menstrual materials should also be provided to ensure reusables can be dried thoroughly during heavy rain after cyclones. 

As noted by key informants, young people face additional challenges in achieving menstrual health than people without disabilities in everyday life. This finding is recorded globally in a growing body of evidence, including Vanuatu [5,6,7,8,9]. These challenges are heightened during emergencies. Hence, integrating holistic menstrual health interventions (which include information, education, and the WASH services to facilitate menstrual health) for young people and their caregivers in times outside emergencies would be a strategic way to improve menstrual health for these populations during humanitarian crises. As stated by key informants, developing partnerships with disability service providers, Organisations of Persons with Disabilities, and relationships with people with disabilities outside emergencies would facilitate greater coordination between actors. Such connections could also improve understanding of disability and menstrual health and the delivery of more effective and efficient inclusive menstrual health humanitarian assistance. 

Existing evidence that explores the provision of menstrual health support for people with intellectual disabilities by caregivers in Nepal, Taiwan, and India, notes that female family members provide menstrual health assistance in these contexts [8,18,44,45]. However, of the few studies that analyse menstrual health for people with disabilities in emergencies, one includes a case study from a man in Burundi who supports his sister in managing menstruation [14]. Our analysis also highlights that men might support the menstrual health of young people with intellectual disabilities or be the primary caregiver. Consequently, a primary focus on female caregivers should not exclude male family members, who may take up this role for the first time during an emergency and be more invisible because of prevailing assumptions and socio-cultural beliefs about menstruation. Depicting and including male and female caregivers in menstrual health information disseminated during emergencies could be a first step in challenging gendered assumptions and encouraging more open dialogue about menstrual health. 

In our study, caregivers reported challenges related to providing menstrual health support for young people. These were similar to accounts from caregivers in other settings, such as Nepal, India, and Taiwan, where family members noted feeling overwhelmed and isolated, especially when the young person was menstruating [8,18,44]. Reasons are complex, including a desire to follow menstrual restrictions (such as not entering the kitchen and touching food), fear of unwanted pregnancy, worry about who will provide care when the primary caregiver is unable to, and increased laundry during menstruation. The similarity of findings from these different settings indicates that caregivers may face these challenges in other LMICs. 

Like others (40), our study highlights the joy that caregiving can bring. However, it must be noted that the caregiver who said, “I’m doing this because children such as these bring blessings,” was an exception (or an ‘outlier’) in our study. This caregiver was more educated, financially better off than her counterparts, and had a husband who was present. Following the interview with this caregiver, we explored any potential correlation between caregivers’ perceptions of their role and levels of education and financial poverty. Yet we were unable to find anyone else we could compare. 

Using the doll during the interview was an effective way to engage young people and explore how they practically manage menstruation. The researchers discussed their assumptions with the young person’s caregiver and then wrote up field notes which were analysed. Dolls have been used to teach young people how to manage menstruation and in research to explore menstrual behaviours [16,19]. The use of dolls alongside other participatory methods, such as Talking Mats [46] or Cue Cards [47], should be applied in different settings to facilitate the direct voices of people with intellectual disabilities in research. 

### Study Strengths and Limitations 

A key strength of this study was that the data was gathered by ni-Vanuatu women with and without disabilities, who have professional experience with disability, menstrual health, and qualitative research methods. We administered several qualitative research methods to explore issues directly with young people with intellectual impairments, whose direct voice is often absent from research. The team was guided by an academic who is experienced in researching menstrual health with people with disabilities in Vanuatu and other settings. However, as members of the research team had previously conducted research exploring the menstrual health of people with intellectual disabilities in Vanuatu and Nepal outside emergencies, we could have introduced bias in the results, including the interpretation of data. We managed and minimised this by double-coding transcripts and regularly discussing data analyses and emerging findings with the team. Some key informants were interviewed by research team members they had previously worked with. This existing relationship may have influenced their responses. We managed this by following a detailed informed consent process and reiterating confidentiality and anonymity during interviews. Finally, though our study findings are transferrable, the humanitarian context in Vanuatu does not reflect all emergency contexts, such as armed conflict or refugee camps. Further research is required to understand the situation and experiences of people with intellectual disabilities and their caregivers in such settings.

## 5. Conclusions

This study aimed to explore the menstrual health experiences of women and girls with intellectual disabilities and their caregivers during emergencies in Vanuatu. To the best of our knowledge, it is the first study of its kind, which contributes to filling this significant gap. We found that gender, disability, menstrual stigma, and discrimination overlap in this context to significantly curtail young people’s participation in daily life and their caregiver’s ability to earn an income. As income poverty increases and mental health declines during emergencies, families are less able to recover. Furthermore, a desire for privacy when the young person is menstruating and a need to keep them safe and secure influence and reduce the caregiver’s choices. Menstrual health interventions targeting this population must be delivered throughout all poverty reduction efforts, from disaster preparedness to community development in Vanuatu and other emergency contexts. If people with intellectual impairments and their caregivers remain invisible in disaster preparedness plans, they will fall further into poverty with each disaster that strikes.

## Figures and Tables

**Table 1:** Study population characteristics.

Young Person		Caregiver		Key Informant	
**Characteristics**	**N=**	**Characteristics**	**N=**	**Characteristics**	**N=**
***Age group***		***Gender***		***Organisation type***	
15–18	5	Female	15	International government organisation	2
19–31	12	Male	2	Non-government Organisation	4
***Impairment type***		***Location***		Disability organisation	3
Visual	0	Urban	8	Private sector	1
Hearing	0	Rural	9	***Location***	
Mobility	3			Espirito Santo	2
Cognition	17			Efate (Port Vila)	4
Communication	9			New Zealand	1
Multiple **^1^**	10				

^1^ Multiple included more than one impairment type and self-care limitation (e.g., washing, dressing, toileting).

## Data Availability

The authors confirm that the data supporting the findings of this study are available within the article [and/or] its supplementary materials.

## Supplementary Materials

The following supporting information can be downloaded at: https://www.mdpi.com/article/10.3390/ijerph192114540/s1, S1 In-depth interview topic guide for caregivers, S2 PhotoVoice and ranking, S3 Observation of the young person.
