# Supplementary material for: Menstrual Health Experiences of People with Intellectual Disabilities and Their Caregivers during Vanuatu’s Humanitarian Responses: A Qualitative Study
Source: Int J Environ Res Public Health. 2022 Nov 5;19(21):14540. doi: 10.3390/ijerph192114540 (PMC9653728; doi:10.3390/ijerph192114540)
Supplement: Supplementary file 1 [file ijerph-19-14540-s001.zip › S1_In_depth_interview_topic_guide_caregiver.pdf]

# Topic Guide: Caregiver

Washington Group Short Set Questions (circle the answer)
I'm now going to ask you a few questions about any difficulties the person you care for has (circle the answers)

**Table S1.** ***Washington Group Short Set Questions***

| | **Functional domain** | **No difficulty** | **Some difficulty** | **A lot of difficulty** | **Cannot do it at all** |
|---|---|---|---|---|---|
| A | Does [name] have difficulty seeing, even if they are wearing glasses? | 1 | 2 | 3 | 4 |
| B | Does [name] have difficulty hearing, even if they are using a hearing aid? | 1 | 2 | 3 | 4 |
| C | Does [name] have difficulty walking or climbing steps? | 1 | 2 | 3 | 4 |
| D | Does [name] have difficulty remembering or concentrating? | 1 | 2 | 3 | 4 |
| E | Does [name] have difficulty with self-care such as washing all over or dressing? | 1 | 2 | 3 | 4 |
| F | Using your usual (customary) language, does [name] have difficulty communicating, (for example understanding or being understood by others)? | 1 | 2 | 3 | 4 |

GUIDING QUESTIONS

| | | |
|---|---|---|
| **OPENING / RAPPORT BUILDING QUESTIONS** | | |
| 1 | **Can you tell me a bit about yourself? Do you live here with others? Tell me about them.** | • Do you work / go to school? What work do you do / where do you go to school? |
| 2 | **What is your relationship to <name of person with a disability>** | • How long have you been supporting her? |
| 3 | **Do you help <name> with daily activities, like washing and eating?** | • Explore WASH related issues [e.g. collecting water, self-care, including bathing, MHM, going to the toilet]? |
| **UNDERSTANDING DISABILITY** | | |
| 4 | **How old is <name>?** | • Can you tell me about her disability?<br>• How long has she had her disability? |
| **HUMANITARIAN CONTEXT** | | |
| 5 | **Were you affected by <Cyclone Pam, Ambae evacuation, Cyclone Harold / other humanitarian crisis]>** | • Can you briefly tell me what happened please?<br>• Probe: if they were internally displaced, where they lived during that time, how long they were living somewhere other than home |
| 5a | *AMBAE EVACUEES:* | • *How long were you in the evacuation centres during the Ambae evacuation?*<br>• *Did you have a choice in staying at the evacuation centre*<br>• *Were you and [name] able to stay together?* |
| 5b | *AMBAE & CYCLONE HARLOD EVACUEES:* | • *How long were you in the evacuation centres during the Ambae evacuation?*<br>• *How long were you in the evacuation centres during Cyclone Harold?* |
| **MENSTRUATION** | | |
| *Now we are going to move on to our discussion about menstruation. This will focus on [name's] experiences, and your experiences of supporting her to manage menstruation. First, I'm going to ask you about what it was like before [humanitarian crisis], and then we will talk about what it was like during and after the [humanitarian crisis] so that I can understand how the situation compares.* | | |

<table>
<tr><td>6</td><td>What are words that girls and women use to talk about menstruation?</td><td>• What words are used with friends?<br>• What do they mean?<br>• Are ‘secret’ words used so nobody else will know what is discussed?</td></tr>
<tr><td>7</td><td>Can you tell me what happened when <name> menstruated <u>BEFORE</u> the <humanitarian crisis>?</td><td rowspan="2">You may need to skip around the topic guide here and probe into the following specific areas:<br>• Knowledge and understanding (Q9)<br>• Behaviour (Q12)<br>• Menstrual materials (Q14)<br>• Changing menstrual materials (Q19)<br>• Disposal / reuse (Q23)<br>• Washing the body (Q25)<br>• Menstrual pain (Q28)<br>• Preparing for menstruation (Q30)<br>• Support / guidance (Q32)<br>• Menstrual restrictions (Q34)</td></tr>
<tr><td>8</td><td>What was it like when <name> menstruated <u>DURING/AFTER</u> the <humanitarian crisis>?</td></tr>
<tr><td colspan="3">KNOWLEDGE AND UNDERSTANDING</td></tr>
<tr><td>9</td><td>Does <name> know what menstruation is? How?</td><td>• Who told her about it (family, friends, teachers, health worker)?<br>• What did they tell her?<br>• Do you need to remind her what it happening every time she menstruates?</td></tr>
<tr><td>10</td><td><u>BEFORE</u> the <crisis> did <name> tell anyone when she was menstruating?</td><td>• If yes, who did she talk with? Why? What did she talk about? Did she try to hide it or keep it a secret? Why?<br>• If no, how did you know when she was menstruating?</td></tr>
<tr><td>11</td><td>Did this change <u>DURING/AFTER</u> the <crisis>?</td><td>• Who did she speak to? Why do you think that was?<br>• What did this mean for you?<br>• How did you respond?</td></tr>
<tr><td colspan="3">BEHAVIOUR</td></tr>
<tr><td>12</td><td><u>BEFORE</u> the <crisis>, did <name’s> behaviour change before or during menstruation?</td><td rowspan="2">• Why do you think this happens?<br>• What does this mean for you?<br>• How do you respond?<br>• Probe into any management solutions the participant mentions (e.g. cuddle her, let her rest, provides pain relief)</td></tr>
<tr><td>13</td><td>Did this pattern change <u>DURING/AFTER</u> the <crisis>?</td></tr>
<tr><td colspan="3">MENSTRUAL MATERIALS</td></tr>
<tr><td>14</td><td><u>BEFORE</u> the <crisis>, what menstrual material did she use (pads, cloth, tissues, underwear, nothing)?</td><td>• Who/where did she/you get materials from?</td></tr>
</table>

| | | |
|---|---|---|
| 15 | **Who/where did she/you get the materials from?** | • Where did she/you hear about that? |
| 16 | **<u>DURING/AFTER</u> the <crisis>, did she continue using the same menstrual materials?** | |
| 16a | ***YES:*** *Skip to question 17* | |
| 16b | ***NO:*** *Why did she use a different material?* | • *How did [name] react to using the different menstrual material? Why?*<br>• *Which material does <u>she</u> prefer using? Why?*<br>• *Which material do <u>you</u> prefer she uses? Why?* |
| 17 | **<u>DURING/AFTER</u> the <crisis>, were you given a hygiene kit and/or menstrual materials?**<br><br>**NO:** skip to next question | • Who/where did you get the hygiene kit from?<br>• What did the hygiene kit include?<br>• Were the menstrual materials suitable?<br>• Was there anything else you would have liked included in the hygiene kit?<br>• Where did she/you hear about the hygiene kit?<br>• Did you pay for them? Did you find this affordable? |
| 18 | **Has <name> ever taken off her menstrual material in front of other people?** | • YES: this can be quite common. Can you tell me about what happens please? Probe into other people's reactions and what the caregiver does to manage.<br>• NO: skip to next question |
| *CHANGING MENSTRUAL MATERIALS* | | |
| 19 | **<u>BEFORE</u> the <crisis> where did she / you change her menstrual material?** | • Could anyone see you/her?<br>• Was there a door with a lock on it? |
| 20 | **Did <name> face any physical difficulties using this place?** | |
| 20a | *YES: What kind of difficulties does she have?* | • *Has anything been done to make it easier to use (e.g. handrail, movable seat etc)?* |
| 20b | *NO: SKIP to question 20* | |
| 21 | **Could anyone see you/her in the place where you/she changes her menstrual material?** | • Was there a door with a lock on it?<br>• What did she/you find good or difficult about the place she changes her menstrual product? |
| 22 | **<u>DURING/AFTER</u> the <crisis>: repeat questions 19-21 (changing menstrual materials)** | |

| | | |
|---|---|---|
| 22a | *AMBAE & CYCLONE HAROLD EVACUEES* | • *Were there any differences between where she/you changed her menstrual materials during the Ambae and Cyclone Harold evacuations? Please explain*<br>• *Probe into accessibility, privacy and safety* |
| *DISPOSAL / REUSE* | | |
| 23 | **<u>BEFORE</u> the <crisis> where / how did she dispose of the used menstrual material?**<br><br>*OR*<br>**<u>BEFORE</u> the <crisis> where / how did she / you wash and dry the used menstrual material?** | • Why did she/you dispose of the materials there?<br>• What did she/you find good or difficult about this?<br><br>*OR*<br>• Why did she/you wash and dry her menstrual materials there?<br>• What did she/you find good or difficult about this? |
| 24 | **<u>DURING/AFTER</u> the <crisis>: repeat question 23 (disposal / reuse)** | |
| 24a | *AMBAE & CYCLONE HAROLD EVACUEES* | • *Were there any differences between where you / she disposed of, or washed and dried the materials during the Ambae and Cyclone Harold evacuations? Please explain* |
| *WASHING THE BODY* | | |
| 25 | **<u>BEFORE</u> the <crisis>, where did she / you wash her body when she was menstruating?** | • Can [name] collect water herself? |
| 25a | *YES: What kind of difficulties does she have?* | • Has anything been done to make it easier to use (e.g. water bought nearer the home or inside the bathing shelter, handrail, movable seat etc)? |
| 25b | *NO: SKIP to question 25* | |
| 26 | **Did she/ you find anything good or difficult about the place she washes?** | • Please explain |
| 27 | **<u>DURING/AFTER</u> the <crisis>: repeat questions 25-26 (washing the body)** | |
| 27a | *AMBAE & CYCLONE HAROLD EVACUEES* | • Were there any differences between your experiences during the Ambae and Cyclone Harold evacuations? Please explain |
| *MENSTRUAL PAIN* | | |
| 28 | **<u>BEFORE</u> the <crisis> did <name> have menstrual discomfort?** | |
| 28a | *YES: How did she / do you manage the pain (e.g. massage, warm cloth, paracetamol)?* | • *Did she have pain relief? Who? What do they give you?* |

| | | |
|---|---|---|
| | | • *Did this help? If not, how does she behave when she has menstrual discomfort?* |
| 28b | *NO: SKIP to next question* | |
| 29 | **<u>DURING/AFTER</u> the <crisis>, did <name's> menstrual cramps get worse?** | • Did you talk to anyone about this / seek help about this?<br>• How did she/you manage the discomfort? |
| 29a | *NO: SKIP to next question* | |
| *PREPARING FOR MENSTRUATION* | | |
| 30 | **Does <name> know when her menstruation is coming**<br>***OR***<br>**Do you know when her menstruation is coming? How?** | • Does she / you prepare for the menstruation? How?<br>Does she / you know when it has started and finished? How? |
| 31 | **<u>DURING/AFTER</u> the <crisis>, were you able to prepare for her menstruation?** | • NO: why and what made it difficult?<br>• YES: Did the way / she prepare for menstruation change?<br>• How and why? |
| *SUPPORT / GUIDANCE* | | |
| 32 | **<u>BEFORE</u> the <crisis>, did you ever talk to anyone about how to manage [name's] menstruation?** | • Why did you talk to that person?<br>• What did they say?<br>• Did they give you any support or guidance? Was this helpful? |
| 32a | *YES: Who and why did you speak to them?* | • *What support / guidance did you get?*<br>• *Who provided this?*<br>• *Did you find it helpful or not?*<br>• *Did you do anything differently after you had that information?* |
| 32b | *NO: have you ever spoken to anyone else about managing [name's] menstruation?* | • *Who have you spoken to?*<br>• *What did they say?*<br>• *Have you ever considered talking to a pharmacist, nurse, doctor?*<br>• *What information or support would you like to have?* |
| 33 | **<u>DURING/AFTER</u> the <crisis>: repeat questions 32-32b (support/guidance)** | |
| 33a | *AMBAE & CYCLONE HAROLD EVACUEES* | • Were there any differences between your experiences during the Ambae and Cyclone Harold evacuations? Please explain |
| *MENSTRUAL RESTRICTIONS* | | |
| 34 | **Is there anywhere [name] is not allowed to go when she is menstruating?** | • Are there restrictions placed her? Mobility? Cooking? Eating? Religious? Why?<br>• Are there things she prefers not to do? |

| | | |
|---|---|---|
| | | • Does she leave the house? Where does she go (work, school, friends)? |
| 35 | **Has this changed <u>DURING/AFTER</u> the <crisis>?** | • How, what why?<br>• How have you / she responded to those changes? |
| *MENSTRUATION AND INCONTINENCE* | | |
| 36 | **Now I'm going to ask you a few questions about <name's> toileting. Are you happy for me to continue?** | • YES: ask next question.<br>• NO: skip to Biggest Challenges, Q39 |
| 37 | **Is <name> able to hold onto her urine and faeces?** | • YES: ask next question.<br>• NO: skip to Biggest Challenges, Q39 |
| 38 | **<u>BEFORE</u> the <crisis>, how did she / you manage that?** | |
| 39 | **Has this changed <u>DURING/AFTER</u> the <crisis>?** | • How, what why?<br>• How have you / she responded to those changes? |
| 39a | *AMBAE & CYCLONE HAROLD EVACUEES* | • Were there any differences between your experiences during the Ambae and Cyclone Harold evacuations? Please explain |
| *BIGGEST CHALLENGES* | | |
| 40 | **<u>BEFORE</u> the <crisis>, what did you feel was the biggest challenge when <name> menstruated?** | • Why is that the biggest challenge? |
| 41 | **<u>DURING/AFTER</u> the <humanitarian crisis>, what did you / <name> find most challenging when she was menstruating?** | • Why is that the biggest challenge?<br>• Did you speak to anyone about this, or seek support anywhere? |
| 41a | *AMBAE & CYCLONE HAROLD EVACUEES: ask question x and sub questions separately* | • i.e. What did you / <name> find most challenging when she was menstruating during the Ambae evacuation? Why? |
| 42 | **<u>DURING/AFTER</u> the <humanitarian crisis>, was there anything, related to menstruation, that was better?** | • Why was this? |
| **RECOMMENDATIONS** | | |
| 43 | **In your opinion, <u>DURING/AFTER</u> the <crisis>, what do you think could be done to:** | 1. Help <name> manage her menstruation better<br>2. Help you support <name> during her menstruation |
| 44 | **Thank you very much for talking to me about your experiences. I have a few more short questions for you, but before I ask those, is there anything else you want to tell me? Is there anything you would like to ask me?** | |
