# Supplementary material for: Menstrual Health Experiences of People with Intellectual Disabilities and Their Caregivers during Vanuatu’s Humanitarian Responses: A Qualitative Study
Source: Int J Environ Res Public Health. 2022 Nov 5;19(21):14540. doi: 10.3390/ijerph192114540 (PMC9653728; doi:10.3390/ijerph192114540)
Supplement: Supplementary file 1 [file ijerph-19-14540-s001.zip › S2_PhotoVoice_and_ranking.pdf]

Topic guide: PhotoVoice and ranking

First meeting

PhotoVoice Process

This first meeting will take up to one hour.

1. **Seek initial informed consent. If granted, continue**

2. **Understanding the camera** - A photographic camera (digital), or a camera phone will be provided to the participant. The researcher should explain simply how the device works and allow the participant to observe (or feel if they are visually impaired) where the lens is and how the shutter works (a visually impaired person can listen to the shutter noise and feel the lens while closed, so not to leave marks on the lens). The researcher should take time to teach the participant the basic features of the device including how to switch the camera off and on, how to take a photo and how to view the photo, how to change the battery. Allow the participant to have a go taking a photo to see what it feels like. It is important that while the participant is learning about the camera they are also holding it so that they begin to be familiar with how it feels. This is particularly important for people with sensory impairments so that they begin to learn how the buttons feel and how they are positioned. A useful starting point is to remind the individual to always use the wrist strap/head strap.

3. **Understanding photography** - Since many of your participants may never have seen a camera before and may have seen relatively few photos in their life, it is also important to explain the purpose of photography. A simple way to explain this is to say that photography can serve several purposes. You can use photography to capture a moment you want to remember as if it was real again. Give an example of this by getting the participant to take a photo of the researchers or their house, view the photo (on the device) and point out that it looks exactly like it is in real life. Then explain that you can also use photography as if it was art by arranging things in a certain way that tells a story or creates a version of reality that can be explored or questioned. Give an example of this too – if you want to take a photo of the idea of 'hunger' it may be hard to show this literally. But you could use symbolism to show hunger. You might have a family seated in their living room, all looks normal except all of them have empty bowls in front of them. Explain that what we are going to do today is use photography to tell their story and creatively express their views.

4. **Understanding the elements of Photography** –
   a. **Taking photos without showing a face to protect the participant's identity** – people may wish to hide their own and other's identity, so you will need to explain how to do this. Techniques include:
      i. Taking photos with the light behind the subject so the figure will be in shadow.
      ii. Focusing on something behind or in front of the subject. This means that the subject will be in soft focus
      iii. Photographing a person's shadow
      iv. Taking a photo of someone from behind (the back of their head, their head / body etc)
      v. Not taking photos of their own house

b. **Ensuring photos protect the participant's dignity** – explain to the participants that any photos of MHM 'accidents' will not be used. This includes clothes, bedsheets or other materials with blood, faeces or urine stains on it
c. **Landscape/Portrait -** Shooting can be done vertically (portrait) or horizontally (landscape). Show participants how this affects the image and explain that portraits can be better when you are focusing on a person and landscapes can be better when you want to capture more of the environment. For people who are visually impaired this can be demonstrated using a mount board window, which can be rotated and felt by the participants. A collection of tactile objects such as toys or fruit can be a good focus for this exercise – the window can be placed by the display in each position and the difference in what is contained in the 'photo' felt through the window.
d. **Framing -** Explain that when taking a photograph it is not simply a matter of pointing towards the subject, but of deciding what is included in the photo – all or some of the subject, the subject and the background, the subject and what is above it etc. Tactile objects can be a useful reference for explaining this concept to someone who is visually impaired. Show the participant how to adjust the framing by using the zoom.
e. **Foreground/background -** This must be explained in a verbal way as well as using their body as a reference. For example, you can ask two participants to stand one in front of the other, and then explain who is in the foreground and who is in the background and what that would mean in a photograph (i.e. who would seem more important, more prominent, larger in the frame etc). Show the participant how to change the focus on the camera.
f. **Distance (only for people with visual impairments) -** When taking a photograph, it is very important to identify the distance to the subject, in order to be sure that it is framed as desired. This can be done by reaching with or laying out a cane, measuring it with steps, or measuring with the joints, such as hands, wrists, arms and forearms. It can be very reassuring for a photographer to know how a photo of a person will be framed if taken from the distance of one cane's length, for example.
g. **Focus/blur -** It is important to clearly identify the area that needs to be in focus. The photographer needs to remember that she can communicate different feelings or ideas depending on what is focused on in the photograph. Here is one way the concept can be explained in a way that makes sense to someone with no sight: When one touches a glass bottle, one identifies the material, its temperature, its dimensions and every detail that makes one recognize the object as a bottle. If this is done again with a thin cloth over the bottle, the details of the bottle won't be recognized so precisely. Nevertheless, one will still know it is a bottle, since some details, like its shape and size, are still recognised. This is what happens when one sees an image that's blurry or out of focus; one recognizes what it is but cannot make out the details clearly.
h. **Light -** Light plays an important role in a photograph since it produces different effects, which lead to different feelings in the observer. A person in darkness, for example, may convey an experience of feeling hidden, where as a person in bright light may convey confidence or nothing to hide. These effects need to be explained fully to blind or visually impaired photographers who will not necessarily realise the impact of the light on their work. To explain this, try to invite the participant to think about the warmth they feel on their face if they are in the sun and use this sensation to determine where the light is coming from. Also teach the participant how to use the flash setting for dark environments.

5. **The photographic task** - Once the participant is comfortable with all this, set them their task. Take five photos of things in their day that makes them happy. Then take five photos that represent their feelings about the person with a disability's menstruation and how they supported that person to manage it during the [humanitarian crisis]. Explain that it is their choice about what they photograph. If there is anything they do not want to photograph (i.e. the menstrual product they use), they do not have to. Ask the participant if anything springs to mind. Help them make a list of the important issues. Ask the participant how they would capture this issue and their feelings around it in a photo. If the participant needs more guidance, work through one example with them, but make sure they lead the process. Stand back whilst the participant takes the photos. Offer guidance, but don't lead them to take specific photos

6. **Self-Directed portraits** - It is likely that in settings where participants are unfamiliar with cameras and photography that they will be keen to be in the photos rather than just taking them. If the participant wants to be in the picture then they still had to direct the field researcher as to how they wanted the photo to look, providing direction on whether it was to be a portrait or landscape shot, what was in the foreground or background, how much of their body should be in shot etc.

7. **Arrange a suitable time to return to the individual's house to give them their photos and have a discussion.**

8. **Save the photos on an encrypted device and send the link to the team leader for review.**

9. **Arrange a time to discuss the photos.**

Second meeting

Interview and interview questions

- This second meeting will take up to one hour
- Run through the information sheet (you will seek their secondary consent and complete the consent checklist at the end of the interview)
- Show the participant the printed photos
- Discuss each one, following the guiding questions below
- Ask the participant to rank the photos according to which they feel is the most important issue to the least important issue
- Seek final consent to use the photos by going through the consent checklist
- It is impossible to do guiding questions for PhotoVoice as we don't know what participants will take photos of. Have the copy of the interview packs for MHM with you. If any of the photos related to any of the topics explored in the interview pack, draw on the relevant questions.

INSTRUCTIONS: INTERVIEWER TURN ON THE TAPE RECORDER AND SAY CLEARLY THE DATE, TIME, LOCATION, AND THE INTERVIEWER'S NAME

- Go through one photo at a time. Ask all these questions for each photo

1. **We have [insert number] photos you took which shows issues related to [name's] menstruation and how you support her to manage. Which photo shall we talk about first?**

2. **What is this photo of?**
   - What story are you telling when taking this photo?
   - Why did you take this photo?
   - Why is this important to you?
   - Refer to the MHM topic guide for more questions

3. **What caption would you like to come up with for this photo?**
   - *To explain what a caption is, ask the participant to imagine that there is a person looking at the photo who doesn't know them or anything about how they live – their task is to explain to them the experience they were trying to convey in two to five words.*
   - *You may need to help the participant with this, but don't put words into their mouth*

When you have discussed each photo:

4. **Now I'd like you to rank your photos from the least important to most important. Please put the least important issue on the left, and work towards the most important on the right**

   - *Take a photo of the ranking, or write the order in your notebook*

5. **Why have you ordered them this way?**

- What makes this the most important issue?
- Run through reasons for the order of all the photos
- Take a photo of the order

6. **Those are all of our questions. Do you have anything else you want to say to us?**

7. **Thank you so much for the time you have taken with us. What you have told us will really help us, and others understand the issues faced by people like you. Then we can try and develop approaches to better assist people like you.**
    - Leave the printed photos with the participant

8. **Go through the consent checklist with the participant**

9. **Name the photos (with the name of the photographer and its order in the ranking)**

10. **Save them on an encrypted device and then send the link to the lead researcher**
