# Supplementary material for: Menstrual Health Experiences of People with Intellectual Disabilities and Their Caregivers during Vanuatu’s Humanitarian Responses: A Qualitative Study
Source: Int J Environ Res Public Health. 2022 Nov 5;19(21):14540. doi: 10.3390/ijerph192114540 (PMC9653728; doi:10.3390/ijerph192114540)
Supplement: Supplementary file 1 [file ijerph-19-14540-s001.zip › S3_Observation_of_the_young_person.pdf]

Topic guide: observation of persons with intellectual disabilities

Guidance

Nb. The note taker should be constantly taking notes about what the participant does, how she behaves and reacts. Capture as much detail as possible.

No.	Instruction	Question to the participant
**Introducing the doll**		
1	Show the participant the doll. Keep her fully clothed. Pass the doll to the participant	
	Note taker: *observe and record the participant's reaction*	
2	Take the doll back. Show the participant that she has removable clothes by taking her top and skirt/trousers off. Leave her underwear on. The participant can take the doll and look at it again if she'd like.	
**Removing a used menstrual product**		
3	Holding the doll, take her underwear, with the used menstrual product in them off	
	Note taker: *observe and record the participant's reaction*	
4	Pass the used menstrual product and doll to the participant	
	Note taker: *observe and record the participant's reaction. What reaction does she have? Is she shocked/disgusted/unmoved? Will she touch the menstrual product?*	
5	Pass the used menstrual product to the participant	
	Note taker: *observe and record the participant's reaction. What reaction does she have? Will she touch it? Will she look at it?*	
**Disposing of a used menstrual product** [have the menstrual bin and the clean menstrual product next to you, in the participant's line of sight]		

6	Take the doll back	What should she [doll] do with the used menstrual product?
	Note taker: *observe and record what the participant does*	
7	If she puts it in the menstrual bin	What should she [doll] do now?
	Note taker: *observe and record what the participant does*	
8	If she does not know what to do, move the bin closer to the participant. If she doesn't put the product in the bin, observe where she puts it	
	Note taker: *observe and record what the participant does*	
**Putting a new menstrual product in the doll's underwear**		
9	Make sure the new menstrual product is beside you and in sight of the participant	What should she [doll] do next?
	Note taker: *observe and record the participant's reaction*	
10	If the participant doesn't point to, or pick up the new menstrual product, pass it to her	
	Note taker: *observe and record the participant's reaction*	
11	If the participant does not know what to do, put the new menstrual product in the doll's underwear	Can I show you?
	Note taker*: make note of this*	
12	If the participant puts the new menstrual product in her underwear, give her a lot of encouragement	
	Note taker*: make note of this*	
**Pain relief for menstrual discomfort**		
13	Put the pained expression on the doll. Bend the doll over and put her hands on her stomach (like she is doubled over with pain)	What do you think is wrong with her [doll]?
	Note taker: *write down what she says/does*	

14	If the participant doesn't know	Do you think she might have menstrual cramps/pain?<br><br>Do you have menstrual cramps?
15	If the participant says she does have menstrual cramps	Can you point to where you feel pain on your body or the doll's body?
	Note taker: *write down what she indicates. If she doesn't have pain, write that down*	
16	If she does have pain, say that this is completely normal	
17	If she has pain ask:<br><br>She may say sleep, drink warm water, put a hot water bottle on her stomach, hug her carer. Try not to suggest these things as she may just say 'yes' to all of them. We will be asking the carers similar questions so will get information from them	What do you do when you have pain?
	Note taker: *write down what she explains or indicates*	
18	Take the pained expression off the doll's face. Say that's all your questions and you've enjoyed spending time with the participant. Ask her if there's anything she wants to tell you. If not, thank her and close the interview	
